# Antimicrobial Resistance Risk Assessment Models and Database System for Animal-Derived Pathogens

**DOI:** 10.3390/antibiotics9110829

**Published:** 2020-11-19

**Authors:** Xinxing Li, Buwen Liang, Ding Xu, Congming Wu, Jianping Li, Yongjun Zheng

**Affiliations:** 1 Beijing Advanced Innovation Center for Food Nutrition and Human Health, College of Information and Electrical Engineering, China Agricultural University, Beijing 100083, China; lxxcau@cau.edu.cn (X.L.); S20193081376@cau.edu.cn (B.L.); 2 Beijing Advanced Innovation Center for Food Nutrition and Human Health, College of Engineering, China Agricultural University, Beijing 100083, China; 386048263@alu.cau.edu.cn (D.X.); ljping@cau.edu.cn (J.L.); 3 College of Veterinary Medicine, China Agricultural University, Beijing 100083, China; wucm@cau.edu.cn

**Keywords:** drug resistance, microbial, database system, risk assessment, DRI, second-order Monte Carlo method

## Abstract

(1) Background: The high use of antibiotics has made the issue of antimicrobial resistance (AMR) increasingly serious, which poses a substantial threat to the health of animals and humans. However, there remains a certain gap in the AMR system and risk assessment models between China and the advanced world level. Therefore, this paper aims to provide advanced means for the monitoring of antibiotic use and AMR data, and take piglets as an example to evaluate the risk and highlight the seriousness of AMR in China. (2) Methods: Based on the principal component analysis method, a drug resistance index model of anti-*E. coli* drugs was established to evaluate the antibiotic risk status in China. Additionally, based on the second-order Monte Carlo methods, a disease risk assessment model for piglets was established to predict the probability of *E. coli* disease within 30 days of taking florfenicol. Finally, a browser/server architecture-based visualization database system for animal-derived pathogens was developed. (3) Results: The risk of *E. coli* in the main area was assessed and Hohhot was the highest risk area in China. Compared with the true disease risk probability of 4.1%, the result of the disease risk assessment model is 7.174%, and the absolute error was 3.074%. Conclusions: Taking *E. coli* as an example, this paper provides an innovative method for rapid and accurate risk assessment of drug resistance. Additionally, the established system and assessment models have potential value for the monitoring and evaluating AMR, highlight the seriousness of antimicrobial resistance, advocate the prudent use of antibiotics, and ensure the safety of animal-derived foods and human health.

## 1. Introduction

With the development of its economy, China’s animal-derived food output has continued to increase. In 2018, China’s animal-derived food output reached 21,387 million tons, but the rapid increase in production has been accompanied by the widespread use, of antibiotics [1], which has made the issue of antimicrobial resistance (AMR) increasingly serious, as it poses a substantial threat to the health of animals and humans [2,3,4]. By 2030 nearly 10 million people worldwide will die each year due to AMR [5,6].

The World Health Organization (WHO) has repeatedly emphasized the serious situation of AMR and advocated the prudent use of antibiotics [7,8]. Additionally, many developed countries have established the standard AMR monitoring system for animal-derived pathogens, including Denmark in 1995, the United States in 1996, etc. [9,10,11,12]. In addition to systems at the national level, small-scale monitoring by AMR systems exists in many hospitals, laboratories, and farms [13,14,15,16]. However, in China, AMR monitoring started relatively late; the first system was not established until 2009 [17], there remains a certain gap in the AMR system between China and the advanced world level. Although the system can upload and download monitoring data in China, it does not have powerful visual query and analysis functions, including monitoring site query, drug resistance rates query of different regions, years, bacteria and animals, drug resistance spectrum, multidrug resistance, and other query functions. In addition, the bacterial monitoring and antibiotic susceptibility testing method in China should be close to or the same as international methods. Additionally, as the amount of data increases, the performance of the system needs to be improved and optimized.

Without powerful analysis functions and models, it is difficult to realize the evaluation and analysis of the AMR problems [18,19]. In this paper, two models are introduced for the achievement of risk assessment; the first is a drug resistance index (DRI) model, and the other is a disease assessment model.

(1) DRI Model

Drug resistance index (DRI) has been developed as an easily understood method and indicator for the assessment of resistance. It creates a single indicator that encapsulates the multiple relationships between pathogen susceptibility and antibiotic use into an easy-to-understand metric that represents the overall level of resistance, and can be performed in both the temporal and spatial domains [20].

Chen and Hughes established DRIs by studying the resistance ratios of microorganisms to antibiotics, evaluated the effects of treating the main pathogenic bacteria, and determined the priority of drug use in hospitals [21], [22]. Ciccolini analyzed the use weights and frequencies of nearly 20 antibiotics for the treatment of *E. coli* and *Klebsiella*, and, based on an exponential method, established a DRI by which to assess the appropriateness of antibiotics for patients [23]. Cornejo assessed antibiotic residues in poultry eggs from backyard production systems in Chile [24].

Collignon examined the relationships between AMR and its potential influences based on a logistic regression model of multivariate analysis, and predicted the resistances of *E. coli* to multiple drugs [25].

However, simply using the antibiotic frequency or cure rate to establish a resistance index is not very reliable [26]. Humans and animals cannot fully absorb the antibiotics they take, which causes a substantial amount of antibiotics to be discharged into the environment, such as in soil and rivers [27], [28]. The impacts of these factors must therefore be considered when establishing DRIs [29]; additionally, DRIs are mainly used for humans, and are rarely established specifically for animals.

In view of these problems, a DRI of anti-*E. coli* drug risk status for animal-derived *E. coli* was established in this work. Based on the PCA method, taking *E. coli* as an example, an anti-*E. coli* drug resistance index model is established to evaluate the drug risk status in China. It innovatively took into account the antibiotic content in the water, soil, and sediment, which was a macroperspective to discuss the serious situation of drug resistance in China.

(2) Disease Assessment Model

Research has shown that the probability of disease can be predicted by studying the relationship between AMR and bacterial pathogenicity. For example, Chitanand indexed multidrug resistance to identify high-risk pollution points in an aquatic environment based on bacteriological analysis [30]. Pouillot assessed the risk of waterborne cryptosporidiosis in France based on a second-order Monte Carlo simulation [31]. Beaudequin summarized a quantitative microbiological risk assessment based on a Bayesian model [32]. Enting assessed the presence or absence of disease-associated factors on growing and fattening pig farms [33].

To achieve a risk assessment, a disease assessment model for piglets was established in this work to predict the probability of *E. coli* disease within 30 days of taking florfenicol based on the second-order Monte Carl, which is of great significance for the early detection treatment, of animal diseases are of great significance and evaluate the effectiveness of antibiotics in the breeding industry.

In summary, this study developed a professional database system for the collection and query of AMR data, and established the DRI model and disease risk assessment model to assess the risk of AMR. The remainder of this paper is organized as follows. In Section 2, the proposed DRI model based on PCA is introduced in detail. In Section 3, a disease risk assessment model based on the second-order Monte Carlo methods is introduced. In Section 4, the proposed system is designed and shown. Finally, in Section 5, final conclusions are discussed.

## 2. DRI Model

A DRI of anti-*E. coli* drug risk status in various regions of China for animal-derived *E. coli* was established; the PCA method was employed based on the antibiotic data in soil and rivers and the resistances of different drugs.

### 2.1. Materials and Methods

#### 2.1.1. Data

This model takes into account not only the antibiotic resistance of *E. coli* in animals, but also expert opinions, literature evidence, data availability, and the environmental effects (air, soil, and moisture) on drug resistance assessment [34]. The AMR data of *E. coli* in pigs in 2013 was used to construct the DRI of each antibiotic. The following describes the data collection methods and data sources.

(1) Data on the use of 36 antibiotics in different regions of China and the environmental concentrations of antibiotics in 58 watersheds in 2013 [35] were included.
(2) At present, this system collects animal AMR data at 892 monitoring sites across the country, covering 26 provinces. The AMR data was determined by minimum inhibitory concentration [36].
(3) The data of pigs released and stored in 31 provinces in 2013 were collected by the China Statistical Yearbook. Due to the areas included in the data do not coincide completely, only the minimum scope of 26 provinces was selected for research, the AMR data of multiple monitoring stations in each province were averaged, and the resistance rates of *E. coli* to the selected 6 drugs in each province were ultimately obtained.

#### 2.1.2. Methods

PCA is a weight calculation method that is not subject to human subjective influence. Employing the concept of dimensionality reduction, it transforms parametric variables into reduced variables and decreases them in order of variance. Throughout this process, the total variance remains unchanged; the largest variance of the first variable is the first principal component, the second variable is the second principal component, etc., and the variance decreases. The first m variables contain most of the information of the variables, and therefore these m variables are ultimately represented as the principal components of the original variables [37].

Therefore, PCA is suitable for processing high-dimensional biological drug resistance information; it is able to combine AMR data, drug data, and environmental factor data, and was used to successfully establish the DRI proposed in this paper. The mathematical model and steps of the PCA method are as follows [38]:

(1) $$\{\begin{array}{c} F_{1}=\alpha_{11}ZX_{1}+\alpha_{21}ZX_{2}+\cdots +\alpha_{p1}ZX_{p}\\ F_{2}=\alpha_{12}ZX_{1}+\alpha_{22}ZX_{2}+\cdots +\alpha_{p2}ZX_{p}\\ \cdots \\ F_{n}=\alpha_{1m}ZX_{1}+\alpha_{2m}ZX_{2}+\cdots +\alpha_{pm}ZX_{p} \end{array},$$

where $\alpha_{1i},\alpha_{2i},\cdots ,\alpha_{pi}(i=1,\cdots ,m)$ is the eigenvector corresponding to the eigenvalues of the covariance matrix ∑  of X, $ZX_{1},ZX_{2},\cdots ,ZX_{p}$ is a value converted by the original variable normalization and needs to meet the $F_{1}$, $F_{2},$ and $F_{n}$ uncorrelated conditions, i.e., the covariance is 0. Therefore, $F_{1}$ is the largest difference in all linear combinations as the first principal component, $F_{2}$ is the second-largest variance as the second principal component, etc.

Standardization formula for the raw data:

(2) $$z_{ij}=\frac{x_{ij}-{\bar{x}}_{ij}}{\sigma_{ij}},$$

where $\sigma_{ij}$ is the standard deviation of $x_{ij}$, and ${\bar{x}}_{ij}$ is the mean of $x_{ij}$. The normalization matrix refers to Equation (3):

(3) $$Z=\left[\begin{array}{cccc} Z_{11} & Z_{12} & \cdots & Z_{1n}\\ Z_{21} & Z_{22} & \cdots & Z_{2n}\\ \vdots & \vdots & \vdots & \vdots \\ Z_{t1} & Z_{t2} & \cdots & Z_{tn} \end{array}\right],$$

Calculate for the correlation coefficient matrix:

(4) $$R=\mathrm{cov}(Z)=\left[\begin{array}{cccc} 1 & r_{12} & \cdots & r_{1n}\\ r_{21} & 1 & \cdots & r_{2n}\\ \vdots & \vdots & \vdots & \vdots \\ r_{t1} & r_{t2} & \cdots & 1 \end{array}\right],$$

where $r_{ij}$ is expressed as the correlation coefficient corresponding to $z_{ij}$.

(5) $$r_{ij}=\frac{\sum_{i=1}^{t}\left(z_{ij}-{\bar{z}}_{j}\right)\left(z_{ij}-{\bar{z}}_{i}\right)}{\sqrt{\sum_{i=1}^{t}{\left(z_{ij}-{\bar{z}}_{j}\right)}^{2}\sum_{j=1}^{n}{\left(z_{ij}-{\bar{z}}_{i}\right)}^{2}}},$$

where ${\bar{z}}_{i}$ and ${\bar{z}}_{j}$ respectively represent the horizontal and vertical averages of the normalized matrix Z.

Calculate for the eigenvalues and eigenvectors of the correlation coefficient matrix.

Calculate for the contribution rate and cumulative contribution rate:

(6) $$w_{i}=\frac{\lambda_{i}}{\sum_{i=1}^{n}\lambda_{i}},$$

where $w_{i}$ is the contribution rate, $\lambda_{i}$ is the non-eigenvector, i=(1,2⋯q) , and q is the number of non-negative feature roots.

Calculate for the component load:

(7) $$L_{ij}=\sqrt{\lambda_{i}}a_{ij},$$

where $a_{ij}$ represents the components of the unit vector.

Calculate for the weights.

### 2.2. Results

Chongqing, which is located in the Jialing River Basin, is used as an example. The relevant data is presented in Table 1.

According to the PCA method described in the previous section, the correlation matrix, total variance interpretation, and component matrix of the data were obtained, and are respectively reported in Table 2, Table 3 and Table 4.

As can be seen from Table 2, there were correlations among variables.

As presented in Table 3 and Table 4, the eigenvalues of components 1 and 2 were greater than that of 1 and could explain 92.332% of the total variance; therefore, components 1 and 2 were chosen as the main components.

Finally, the score coefficient of the component matrix was used as the coefficient, and the standardized value of the original variable was used to calculate the scores of all drugs, which are summarized in Table 5. The higher the ranking score of a drug, the more dangerous it is, and the less recommended it is for the treatment of *E. coli* disease.

The preceding subsection discussed the resistance index of a bacterium to a variety of drugs in a region. To further explore the relationship between antibiotic use, the degree of antibiotic pollution, and bacterial resistance in different regions of China, the DRI values of *E. coli* resistance to doxycycline at the city level were calculated, and the data and final ranking are exhibited in Table 6. In the table, the higher the ranking, the higher the risk of drug usage to treat *E. coli*. This index is of great significance for guiding drug use in various regions and for preventing and controlling the AMR of animal-derived pathogens.

On the whole, since the north is the main area for animal husbandry, its drug resistance situation is obviously worse than that in the south. Comparing the main areas of livestock production, Hohhot is the highest risk of drug usage to treat *E. coli*, while Chongqing and Chengdu are not serious, which has a certain relationship with antibiotic medication habits and economy. The economy in Hohhot is developing slowly, with poor conditions, sparsely populated, and inconvenient transportation. The farming in this area is mostly for retail or family, therefore there are some irregularities in the use of antibiotics. However, Chongqing and Sichuan areas are economically developed and densely populated. Animal breeding has a strong technical guarantee, good working conditions, more intensive factories, more strict regulations on veterinary antibiotics, and the AMR is relatively good. Therefore, the DRI model conforms to the actual situation in China and can be used as an important basis for AMR evaluation.

## 3. Disease Assessment Model

During the nursery period, the digestive function of piglets has not yet been fully established, and the piglets have transitioned from weaning to feeding, which endows them with poor disease resistance. Piglets are directly in contact with a sow or its excrement, thus exposing them to an intestinal infectious disease caused by *E. coli* and its parasitic toxins, which will affect the development of piglets and may even cause death. Florfenicol has been found to cause less damage to animals and is sensitive to chloramphenicol-resistant strains of bacteria and can prevent and treat diseases caused by *E. coli* and other pathogenic bacteria. However, due to the heavy use of this antibiotic, the issue of bacterial resistance has worsened [39].

In this paper, based on the second-order Monte Carlo methods, piglet-*E. coli*-florfenicol models were established during the weaning period of piglets to evaluate the risk degree of using florfenicol to prevent and treat piglet *E. coli* and predict the probability of *E. coli* disease.

### 3.1. Materials and Methods

#### 3.1.1. The Risk Assessment Process

The risk assessment process of nursery piglets during weaning was abstracted into three stages, namely bacterial reproduction, drug therapy, and infection probability, and each stage is subsequently analyzed in detail.

(1) Bacterial Reproduction

This stage primarily assesses the true amount of *E. coli* in a piglet after weaning, thus providing a basis for actual pig morbidity.

Florfenicol resistance in *E. coli* in piglets needs to be detected, and the variable is represented as *R*. Additionally, a uniform distribution for drug resistance is utilized to present the uncertainty.

(8) $$R\left(x\right)=\frac{1}{b-a}\;,a<x<b,a=0.50,b=0.60,$$

During lactation, *E. coli* enters the intestinal tracts of piglets and lies dormant. After weaning, due to the changes in the environment and the change of the feeding mode, the amount of *Lactobacillus* in the piglets will decrease, and the amount of *E. coli* will increase. A normal distribution is used to estimate the intake of *E. coli* in piglets, and the variable is represented as Co with the unit of number/log CFU g^−1^.

(9) $$Co=\frac{1}{\sqrt{2\pi }\sigma }\exp\left(-\frac{{\left(x-\mu \right)}^{2}}{2\sigma^{2}}\right),\mu =5.782,\sigma^{2}=0.33,$$

Although the piglets’ resistance and the feed have a certain inhibitory effect on *E. coli*, the piglets’ environment is very suitable for the growth of *E. coli* without competition; thus, the amount of *E. coli* will increase exponentially. An exponential model was therefore constructed to estimate the amount of *E. coli*, and the variable is represented as *Ct*.

(10) $$Ct=Coe^{-kt},$$

where *k* represents the ability of the piglets’ resistance and the feed to suppress the growth of *E. coli*, and it was set to 0.023 in this work. Additionally, *t* is the growth time of *E. coli* in piglets. To demonstrate the uncertainty, a uniform distribution is used for drug resistance.

(11) $$t=\frac{1}{b-a}\;,a<x<b,a=1,b=30,$$

(2) Drug therapy

This stage primarily assesses the risk of infections in nursery pigs caused by the use of florfenicol and calculates the amount of *E. coli* in piglets after taking the antibiotic.

As antibiotics should not be administered in excess, the number of piglets taking florfenicol must be considered, and the variable is represented as *s*.

(12) s=0.1817t,

It was assumed that piglets were fed every 6 h, and the proportion of *E. coli* that survived after the administration of florfenicol was calculated based on in vitro experiments. The initial amount of *E. coli* in the serum was 3 × 10^7^ CFU·mL^−1^. After the injection of florfenicol, the amount of *E. coli* was found to decrease by 0.47 × 10^7^ CFU mL^−1^ after 1 h, and by 0.63 × 10^7^ CFU mL^−1^ after 6 h. Therefore, the empirical distribution function is used to determine the probability of *E. coli* surviving in piglets after the administration of florfenicol, and it is represented as *i*.

Finally, the amount of *E. coli* in the piglets was calculated and is represented as n and is expressed by the Poisson distribution.

(13) $$P\left(X=k\right)=\frac{\lambda^{k}}{k!}e^{-\lambda },k=0,1,2\ldots ,\lambda =Ct\cdot i\cdot s,$$

(3) Infection probability

This stage primarily calculates the probability of the occurrence of disease in piglets. First, the probability of disease occurrence in normal piglets was determined, as were the amount of *E. coli* and the drug resistance of the piglets. The probability of disease was then calculated based on the second-order Monte Carlo methods.

#### 3.1.2. Methods

The Monte Carlo method is used to transform complex research objects or computational problems into multiple sets of input variables with digital characteristics via scientific statistical modeling; it essentially reduces the computational complexity and then simulates each set of numerical variables to obtain accurate approximate solutions, and fits the probability distribution of its output results [40].

The first-order Monte Carlo simulation method can be used to obtain the empirical distribution of overall risk by reflecting the distribution of the overall parameter variability. The second-order Monte Carlo method, which is based on the first-order Monte Carlo variability, increases the uncertainty, which can be expressed as a larger confidence interval for the model parameters [31]. Disease in piglets is affected by a variety of factors, and drug resistance and environmental uncertainty and variability can contribute to significant differences in the results. The second-order Monte Carlo method can take these uncertainties and variabilities into account, and it is therefore suitable for the assessment of the risk probability of disease.

**Figure 1:**
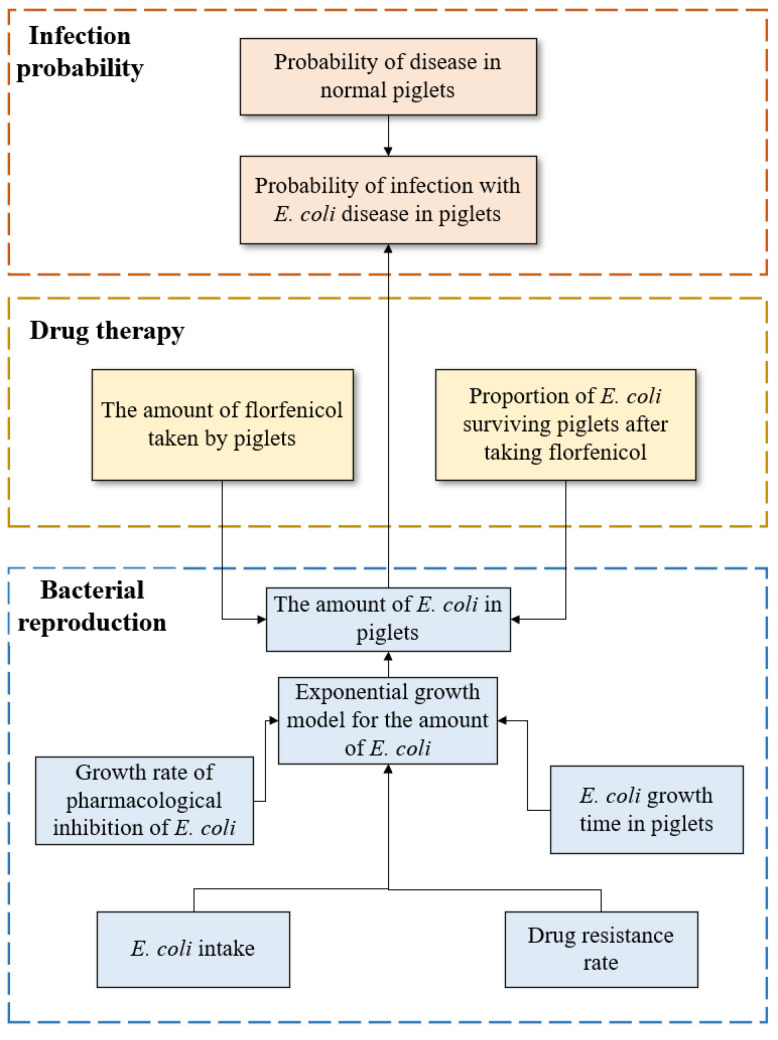
The framework of the second-order Monte Carlo model.

The module of risk assessment was established according to the second-order Monte Carlo method, and it is presented in Figure 1.

### 3.2. Results

The *E. coli* and drug resistance uncertainties and variability of variables that reflect the distribution of overall parameter variability were combined to further obtain an empirical distribution of overall risk. These variables were discussed in the previous section and are exhibited in Table 7.

The second-order Monte Carlo model was executed, and the results are listed in Table 8. In the confidence interval of 2.5–97.5%, the risk value was found to be in the interval [0.00896, 0.1671], and the optimal estimated value after 1001 simulations was 0.07174, which means that the probability of disease after piglets were treated with florfenicol was 7.174%. Compared with the actual result of 4.1%, the absolute error difference was only 3.074%, indicating that the model’s predictive effect was good.

In order to show the prediction results of different geographic regions, based on the available data, we chose to divide China into northeast, north, southwest, and southeast regions, and analyzed the possible disease situation in these regions. Additionally, it is shown in Table 9, it was found that the probability of florfenicol in piglets was different, which was due to the different AMR conditions in these regions.

## 4. System Analysis and Design

### 4.1. System Requirements Analysis

**Figure 2:**
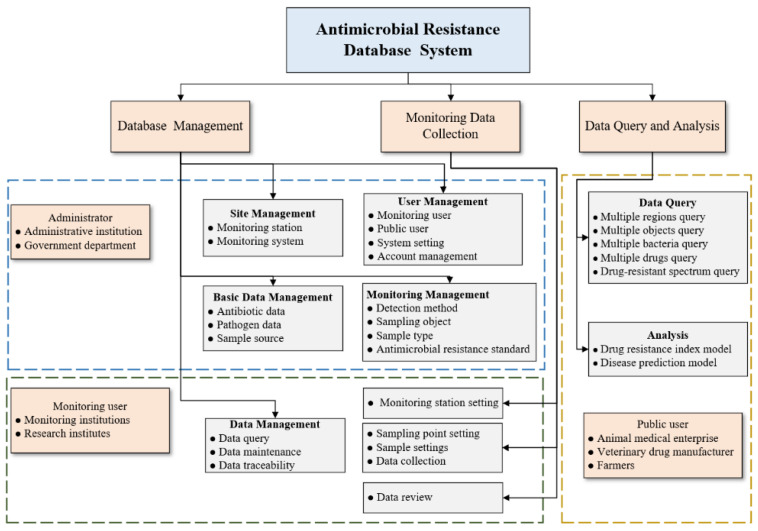
The overall design scheme of the system.

This section analyzed the system requirements based on the current status of China’s AMR monitoring system and actual demand. The overall design scheme of the system is presented in Figure 2.

Users of the system include relevant government departments, monitoring institutions, research institutes, breeding enterprises, medical and veterinary drug manufacturers, etc., and these departments have different needs of the system. Therefore, the system users can be divided into administrative-level users, monitoring-level users, and public-level users.

To guide the monitoring of drug resistance, achieve standardization, and truly assess the risks of AMR, the system should have account management, system configuration management, basic data management, monitoring data collection, AMR data query, and analysis functions. The query function is based on five conditions: the AMR rates, stations, objects, bacteria, and drugs. Additionally, query functions can be realized for the combination of multiple conditions. Moreover, query results are visualized in the forms of charts and histograms. In the analysis function module, the system comprehensively analyzes the resistance status by constructing a visual DRI model and a disease prediction model for piglets, which can reflect the risk of drug resistance and ensure safety.

### 4.2. System Design and Development

The B/S system framework does not require the installation of traditional software, and most of the work is conducted by the server [41,42]. The costs to users and the workloads of system maintenance and management are directly reduced, which results in improved independence, adaptability, scalability, and security [43,44].

**Figure 3:**
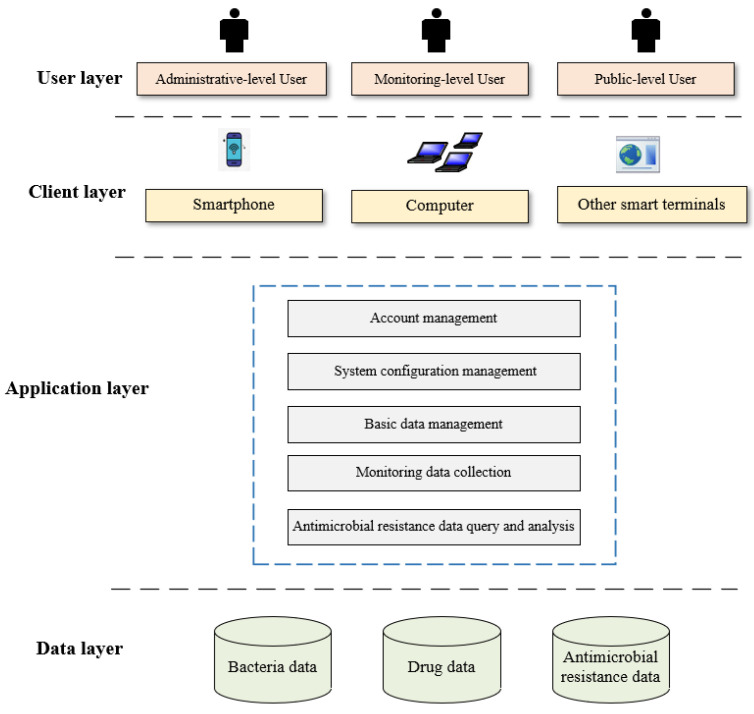
The framework of the system.

Therefore, this system was designed based on the B/S structure; Microsoft Windows Server 2012 Enterprise is used as the background of the web application server, MySQL SERVER 2010 Enterprise is used as the background of the database, and ASP technology is employed. This architecture realizes the connection between the front page and the background database through ADO technology, and various functions are ultimately developed [15]. The framework of the system is illustrated in Figure 3.

### 4.3. The Results of the System

The system classifies its users and can be opened to public users. It also displays the query results of drug resistance data in the forms of bar charts and pie charts and constructs a DRI to evaluate and present a visualization of drug resistance safety. It provides a good data foundation and technical platform for the further analysis of pathogen resistance. The functional tests of the system are presented in the subsequent figures.

**Figure 4:**
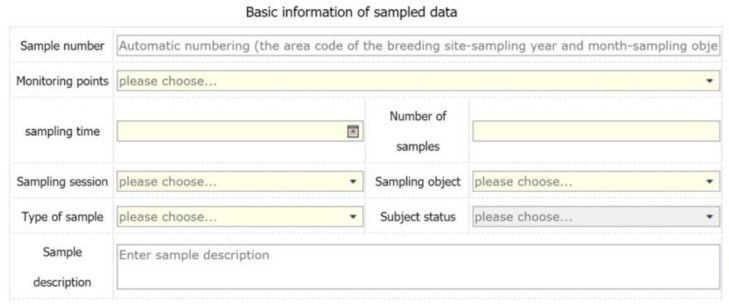
Data collection interface.

Data collection module: this module collects AMR data from monitoring sites across China. The user selects the monitoring site, strain information, drug type, test method, animal breed, sample, and collection time, and uploads the data, as shown in Figure 4. During the data storage process, resistance standards will be automatically compared to update the resistance data, as shown in Figure 4. Additionally, the database system has stored plenty of antimicrobial resistance data in 28 monitoring stations and 892 monitoring points in China.

**Figure 5:**
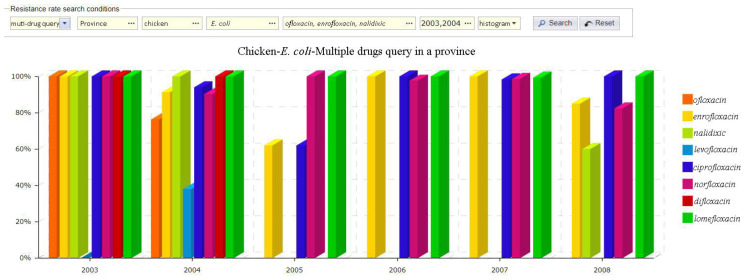
Multiple drugs query (sample data).

Drug resistance rate query module: this module combines the system data with the geographic coordinate information of the monitoring stations to realize the visualization of the spatial distribution of AMR data across China. It realizes the combined query functions of the year, region, animal, bacteria, and drug, and allows for the comparison of drug resistance rates, drug resistance spectra, and multiple drug resistances and which are shown in Figure 5.

**Figure 6:**
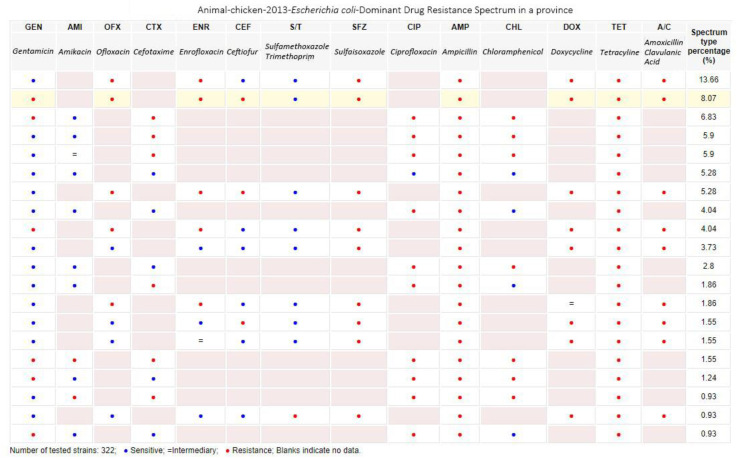
Drug resistance spectrum query (sample data).

Drug resistance spectrum query module: this query provides a statistical results chart of the dominant drug resistance spectra of specific pathogens in each region in a certain year, and the percentages of the combined numbers of test samples, as shown in Figure 6.

**Figure 7:**
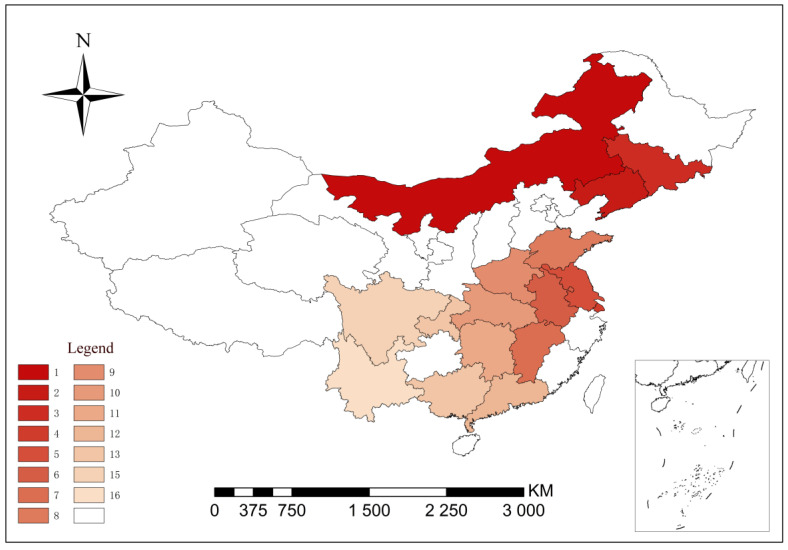
Visual analysis of the drug resistance index (DRI) using doxycycline as an example.

Visual analysis of DRI module: taking doxycycline as an example, the resistance index of *E. coli* was calculated for multiple regions, and the data and the final ranking are presented in Figure 7; the higher the value of a region, the higher the risk that *E. coli* will be resistant to doxycycline.

## 5. Discussion and Conclusions

In this paper, the current research status of AMR monitoring systems and risk assessment models were analyzed, and it was found that there was a large gap between China and developed countries; China has few systems that lack strong risk assessment and analysis functions for animals. In view of the severe situation of AMR and the practical application demands of animal breeding in China, an AMR database system and risk assessment models for animal-derived pathogens were established.

The system and risk assessment analysis models provide advanced and scientific means for the monitoring and assessment of AMR, achieve the collection of data related to AMR in animal breeding, highlight the current seriousness of AMR, raise the public vigilance about AMR, and ensure the safety of animal-derived foods and human health in China. The main contributions and conclusions of this work are as follows.

(1) In this study, a DRI of anti-*E. coli* drug risk status in China was established, which is a comprehensive method for the evaluation of the degree of AMR. The model combines AMR, antibiotic use data, and environmental factors, and reveals antibiotics usage, pollution levels, and the influence of bacterial AMR. It compensates for the shortcomings of the judgment of AMR by only the drug resistance rate and provides support for government decision-making.
(2) In this study, taking *E. coli* and florfenicol as an example, a disease prediction model for piglets based on the second-order Monte Carlo were established to predict the probability of *E. coli* disease. The model can be used for the disease prediction of piglets and were found to achieve good results. As compared with the actual risk probability of piglets of 4.1%, the result of the two-dimensional Monte Carlo model was found to be 7.174%, and the absolute error was 3.074%. The results prove that the models can be applied to the quantitative risk assessment of disease in piglets and provide good evaluation results.
(3) In this study, the AMR data of pathogenic bacteria were classified and summarized according to breeding animals, bacterial species, drug types, test methods, time, etc., and a browser/server (B/S) architecture-based visualization system for animal-derived pathogens was developed. The system realizes the data collection and data management of antibiotics, ensures the integrity of the data chain of the AMR monitoring and analysis of animals, and provides basic data for further AMR prediction and risk assessment.

However, there remain limitations that must be taken into account. One limitation is that although we provided the methods to assess the risk of AMR, due to the lack of data, we only selected piglets as an example. Another limitation is that the prudent use of antimicrobials depends on many criteria that are not considered, including drug levels in the target tissue and the modes of action of antimicrobials (both concentration- and time-dependence). In future studies, more data will be collected to expand the presented system. To improve the efficiency of the proposed method, the estimation of essential criteria will be explored, including animal welfare, accompanying vaccination programs, and pharmacokinetic considerations. It is the hope of the authors that the system will provide both an assessment of AMR and specific guidance for farmers and governments.

## Figures and Tables

**Table 1:** Relevant antibiotic data in Chongqing, 2013.

Antibiotic	Water (ng/L)	Soil (ng/g)	Sediment (ng/g)	*E. coli* Resistance (%)	Drug Usage (t)
sulfamethoxazole	7.36	0	0.01	0.67	5.7
trimethoprim	22	0.09	0.11	0.67	4.52
tetracycline	3.82	0.27	0.75	0.94	3.43
doxycycline	53.3	3.29	19.8	0.97	66.27
ofloxacin	10.2	3.34	4.02	0.19	70.3
enrofloxacin	32.2	8.85	52.5	0.25	89.03

**Table 2:** The correlation matrix of relevant antibiotic data in Chongqing, 2013.

Component	Water (ng/L)	Soil (ng/g)	Sediment (ng/g)	*E. coli* Resistance (%)	Drug Usage (t)
Water (ng/L)	1.000	0.489	0.579	0.182	0.575
Soil (ng/L)	0.489	1.000	0.952	−0.596	0.904
Sediment (ng/g)	0.579	0.952	1.000	−0.393	0.777
*E. coli* resistance (%)	0.182	0.596	−0.393	1.000	0.567
Drug usage (t)	0.575	0.904	0.777	−0.567	1.000

**Table 3:** The total variance interpretation of relevant antibiotic data in Chongqing, 2013.

Initial Eigenvalue				Extracted Sum of Squares of Loads		
Component	Total	Percentage of Variance	Cumulative	Total	Percentage of Variance	Cumulative
1	3.415	68.292	68.292	3.415	68.292	68.292
2	1.202	24.040	92.332	1.202	24.040	92.332
3	0.282	5.636	97.968			
4	0.100	2.006	99.974			
5	0.001	0.026	100.000			

**Table 4:** The component matrix of relevant antibiotic data in Chongqing, 2013.

Component	1	2
Water (ng/L)	0.604	0.750
Soil (ng/L)	0.984	−0.087
Sediment (ng/g)	0.930	0.113
*E. coli* resistance (%)	−0.570	0.786
Drug usage (t)	0.945	−0.026

**Table 5:** The drug score summary in Chongqing, 2013.

Antibiotic	F1	F2	F	Ranking
enrofloxacin	5.7022	−0.4114	4.11	1
doxycycline	1.5418	2.1136	1.691	2
ofloxacin	0.9021	−1.5417	0.266	3
trimethoprim	−2.2267	0.1699	−1.603	4
sulfamethoxazole	−2.6993	−0.4134	−2.104	5
tetracycline	−3.2201	0.083	−2.36	6

**Table 6:** *E. coli* doxycycline resistance index in major cities in China.

City	Air (ng/L)	Water (ng/L)	Soil (ng/g)	Sediment (ng/g)	*E. coli* Resistance (%)	Drug Usage (t)	Ranking
Hohhot	4.38 × 10^−18^	11.3	17.3	6.03	0.6875	41.5696	1
Shenyang	3.28 × 10^−17^	36.8	5.82	52.4	0.5	119.7102	2
Changchun	5.35 × 10^−18^	13.3	1.84	98.1	0.6596	71.6386	3
Shanghai	6.87 × 10^−18^	7.65	15.5	85.5	0.7313	11.1733	4
Nanjing	8.28 × 10^−18^	11.4	14.7	45.8	0.6154	134.4612	5
Hefei	8.28 × 10^−18^	11.4	14.7	45.8	0.1954	131.9544	6
Nanchang	3.18 × 10^−18^	0.94	7.42	10.4	0.3438	136.7885	7
Jinan	2.66 × 10^−17^	302	7.22	143	0.3721	204.139	8
Zhengzhou	2.15 × 10^−17^	9.06	3.71	5.57	0.5404	250.7159	9
Wuhan	1.85 × 10^−17^	8.07	13.1	18.1	0.4348	183.3908	10
Changsha	2.20 × 10^−17^	11.5	6.27	39	0.6154	261.0688	11
Guangzhou	3.98 × 10^−18^	21.2	41.2	142	0.5731	161.5657	12
Nanning	5.55 × 10^−18^	10.6	2.57	8.09	0.45	148.6353	13
Chongqing	1.02 × 10^−17^	32.2	8.85	52.5	0.25	89.0268	14
Chengdu	1.58 × 10^−17^	5.15	12.5	17.2	0.4677	315.1172	15
Kunming	2.63 × 10^−18^	3.85	2.93	7.17	0.4062	139.9818	16

**Table 7:** Uncertainty and variability of variables.

Variable	Nsv	Nsu	Median	Max
Co	1	1001	5.750	6.658
t	1	1001	15.071	29.993
R	1	1001	0.549	0.600
Ct	1	1001	2.175	3.572
i	1001	1	0.210	0.210
S	1	1001	2.738	5.450
N	1001	1001	1.000	14.000
r	1	1001	0.064	0.077

Nsv indicates the size of the variability dimension, and Nsu indicates the size of the uncertainty dimension.

**Table 8:** The results of the model.

Index	Mean	Sd	Min	2.5%	25%	50%	75%	97.5%	Max	Nsv
Median	0.07174	0.0635	0	0	0.0000	0.0637	0.112	0.2100	0.320	1001
Mean	0.07541	0.0618	0	0	0.0285	0.0648	0.111	0.2116	0.320	1001
2.5%	0.00896	0.0233	0	0	0.0000	0.0000	0.000	0.0606	0.136	1001
97.5%	0.16717	0.0971	0	0	0.1044	0.1565	0.235	0.3741	0.507	1001

Note: Sd indicates the standard deviation.

**Table 9:** The results of the model in different areas.

Region	Median	Mean	2.5%	97.5%
Southeast	0.0331	0.0351	0.0039	0.0793
North	0.0717	0.0754	0.0090	0.1672
Southwest	0.0601	0.6333	0.0070	0.1387
Northeast	0.0884	0.0910	0.0111	0.2057
